# Exploring Different Extrapolation Approaches for the Critical Temperature of the 2D-Ising Model Based on Exactly Solvable Finite-Sized Lattices

**DOI:** 10.3390/e27111139

**Published:** 2025-11-06

**Authors:** Daniel Markthaler, Kai Peter Birke

**Affiliations:** 1 Institute for Energy Efficiency in Production, University of Stuttgart, Nobelstraße 12, 70569 Stuttgart, Germany; daniel.markthaler@eep.uni-stuttgart.de; 2 Electrical Energy Storage Systems, Institute for Photovoltaics, University of Stuttgart, Pfaffenwaldring 47, 70569 Stuttgart, Germany; 3 Fraunhofer Institute for Manufacturing Engineering and Automation IPA, Nobelstraße 12, 70569 Stuttgart, Germany

**Keywords:** Ising model, statistical thermodynamics, critical temperature estimation, finite-sized lattice

## Abstract

The fact that the Ising model in higher dimensions than 1D features a phase transition at the critical temperature $T_{c}$ despite its apparent simplicity is one of the main reasons why it has lost none of its fascination and remains a central benchmark in modeling physical systems. Building on our previous work, where an approximative analytic free-energy expression for finite 2D-Ising lattices was introduced, we investigate different extrapolation strategies for estimating $T_{c}$ of the infinite system from exactly solvable small lattices. Finite square lattices of linear dimension *N* with free and periodic boundary conditions were analyzed, exploiting their exactly accessible density of states to compute the heat capacity profiles C(T). Different approaches were compared, including scaling models for the peak temperature $T_{\max}\left(N\right)$ and an envelope construction across the set of C(T)-profiles. We find that both approaches converge to the same asymptotic value and compare favorably to the established Binder cumulant method. Remarkably, a model for $T_{\max}$ with a single model parameter following an N/(N+1)-law provides robust convergence, with a physical analogy motivating this proportionality. Our findings highlight that surprisingly few, but highly accurate, finite-size results are sufficient to obtain a precise extrapolation.

## 1. Introduction

Understanding how critical behavior emerges from finite systems remains a foundational challenge in statistical mechanics. The Ising model [1,2] is structurally simple, yet captures the essential mechanisms of cooperative behavior in lattice systems and therefore continues to serve as a versatile and influential testing ground for theories of phase transitions and critical phenomena. Despite its long history, determining specific parameters of the Ising model in the thermodynamic limit from finite-sized lattices with high accuracy remains a benchmark challenge [3]. A wide array of methods has been developed to determine the critical temperature $T_{c}$ of the Ising model [3,4]. The most common approach is to associate the finite maximum in the heat capacity C(T) of a finite lattice as a proxy for $T_{c}$. From theory, it is known that the heat capacity C(T) for the 2D-Ising model shows a logarithmic singularity $C\left(T\right)\propto \ln|T-T_{c}|$ in the vicinity of $T_{c}$ with a critical exponent of α=0 [5]. Monte Carlo (MC) simulations yield accurate numeric estimates but often at high computational cost for large system sizes [6]. The renormalization group framework provides fundamental insights into universality and scaling laws, yet its practical implementations on complex or finite geometries are technically demanding [7]. Finite-size scaling [8,9], particularly through observables like the Binder cumulant [10] or heat capacity peaks, has become the standard route to extrapolate $T_{c}$ from finite lattices but requires extensive data from multiple sizes to achieve high precision.

Alternatively, exact treatments of small systems offer valuable insight. Kaufman’s solution for periodic boundary conditions (PBCs) [11] and subsequent modern implementations [12] provide access to the density of states (DOS) for small lattices, which in principle allows for the exact evaluation of thermodynamic functions. For Ising systems with free (i.e., open) boundaries (FBC), on the other hand, no such solution approaches have been found to date [13]. Notably, Stošić et al. [14] employed the transfer-matrix (TM) method to obtain the exact DOS for a set of finite squared FBC-Ising systems, using their results to test finite-size scaling predictions. However, the lack of compact closed-form expressions for the partition function of arbitrary finite lattices [15] limits the general applicability of such approaches, and the direct extrapolation of $T_{c}$ based on these exact calculations remains challenging. Recent advances in quantum computing have revived interest in finite-size Ising models [16]. While several methods exist for ground-state energies, direct computation of partition functions is far from routine and remains challenging due to unfavorable qubit scaling with interaction complexity [17]. This shows that even in 2025, there is still a need for the systematic use of exactly solvable small lattices for extrapolation towards the thermodynamic limit.

Building on our previous analytic framework for free energy expressions for finite 2D-Ising systems [18], the current study systematically compares different extrapolation schemes aimed at estimating the critical temperature of the infinite lattice from a minimal set of exactly solvable finite lattices with both free and periodic boundary conditions. In particular, we analyze scaling behavior of heat capacity peak temperatures ($T_{\max}$) across sizes and construct common envelope functions. From theory, we expect $\lim_{N\to \infty }T_{\max}\left(N\right)=T_{c}$ [9], which is why we primarily focus on estimation approaches for $T_{\max}$. For alternative temperature measures, we impose the general requirement that, although they do not have to follow the same scaling behavior as $T_{\max}\left(N\right)$, they must converge towards the same limiting value $T_{c}$ and, ideally, be as simple and robust as possible to calculate. We uncover a surprisingly simple two-parameter scaling law following an N/(N+1)-form, which converges robustly towards Onsager’s exact $T_{c}$, that delivers excellent results even in simplified form with only one adjustable parameter.

This work ultimately aims to demonstrate how minimal yet exact finite-size information can deliver precise critical estimates, offering an efficient alternative to resource-intensive traditional methods and potentially serving as a template for other lattice gas models where analytic solutions are elusive.

## 2. Materials and Methods

In this work, we consider the classical Ising model, based on isotropic nearest-neighbor interactions with uniform coupling constant *J* and two possible values for the spin number of the *i*’th spin $s_{i}=\pm 1$, corresponding to the spin-up and spin-down state. In 2D, for finite-sized rectangular lattices of dimensions $N_{y}\times N_{x}$ (with $N_{x}$ and $N_{y}$ spins placed in *x*- and *y*-direction, respectively), the field-free Hamiltonian reads as (see also [18])

(1a) $$\begin{array}{cc} H_{N_{y}\times N_{x}} & =H_{x}+H_{y}\\ \; & \mathrm{with}H_{x}=-J\sum_{i=1}^{N_{y}}\sum_{j=1}^{N_{x}}\underset{\mathrm{right}\mathrm{neighb}.}{\underset{︸}{s_{i,j}\;s_{i,j+1}}}\mathrm{and}H_{y}=-J\sum_{i=1}^{N_{y}}\sum_{j=1}^{N_{x}}\underset{\mathrm{lower}\mathrm{neighb}.}{\underset{︸}{s_{i,j}\;s_{i+1,j}}} \end{array}$$

(1b) $$\begin{array}{cc} \; & s_{i,N_{x}+1}=0\;\left(\mathrm{FBC}\right)\;\mathrm{or}\;s_{i,N_{x}+1}=s_{i,1}\;\left(\mathrm{PBC}\right),\;\forall i=\left\{1,\ldots ,N_{y}\right\} \end{array}$$

(1c) $$\begin{array}{cc} \; & s_{N_{y}+1,j}=0\;\left(\mathrm{FBC}\right)\;\mathrm{or}\;s_{N_{y}+1,j}=s_{1,j}\;\left(\mathrm{PBC}\right),\;\forall j=\left\{1,\ldots ,N_{x}\right\} \end{array}$$

where FBC and PBC denote the case of free and periodic boundary conditions, respectively. $s_{i,j}$ denotes the spin value of the associated spin at lattice position (i,j). Here, we solely focus on square (N×N) lattices, i.e., $N=N_{x}=N_{y}$. Throughout the paper, *J* will be set to unity, i.e., only the ferromagnetic case (J>0) will be considered.

Calculations are based on the exact representation of the canonical partition function $Z\equiv Z(N_{\mathrm{spins}},T)$ as function of spin number $N_{\mathrm{spins}}=N^{2}$ and temperature *T*, using the density of states formalism: $Z=\sum_{n}\Omega_{n}\;e^{-\beta E_{n}}$. The sum runs over distinct energy levels $E_{n}$ with associated statistical weights, i.e., degeneracies $\Omega_{n}\equiv \Omega \left(E_{n}\right)$ which obey $\sum_{n}\Omega_{n}=\Omega_{\mathrm{tot}}=2^{N_{\mathrm{spins}}}$. For convenience, the inverse temperature $\beta =1/k_{B}T$ will be generally used instead of *T*, where Boltzmann’s constant $k_{B}$ will be set to unity to make the heat capacity $C/k_{B}$ dimensionless. With this convention, β=1/T, and we do not need to distinguish between dimensional and dimensionless variants of *C*, *T*, and β, i.e., $C^{*}\equiv C/k_{B}=C$, $T^{*}\equiv k_{B}T/J=T$ and $\beta^{*}\equiv J/k_{B}T=J\beta =\beta$.

In the case of FBC systems, calculation of the DOS (i.e., the collected distribution of discrete energy levels and weights $\left\{E_{n},\Omega_{n}\right\}$) was performed using the transfer-matrix (TM) method [19,20] for system sizes N={2,…,12}. The TM method was implemented in Python 3.11 using standard libraries (numpy, itertools, collections). Energy contributions of individual rows and row pairs were precomputed and combined recursively to obtain the full DOS. The TM-implementation was validated for small system sizes up to N=5 against brute force calculations where all possible $\Omega_{\mathrm{tot}}$ configurations were systematically generated using the Python itertools-module. Up to a size of N=12, the systems can be calculated in moderate computing time via the TM method on a current laptop without the need for access to specialized high-performance computing infrastructure. For larger system sizes, N={13,…,20}, which were not included for model training but for assessing prediction quality, we used the optimized Pfaffian-based algorithm by Karandashev et al. [21], which evaluates the exact partition function of a given 2D-FBC-Ising lattice at specified β. Since the code (which is freely available [22] ) does not deliver the DOS directly but returns lnZ(β), lnZ(β+Δβ), and lnZ(β+2Δβ) with $\Delta \beta =10^{-5}$, the formalism for calculating the maximum heat capacity ($\beta_{\max}$, $C_{\max}$) presented below is not applicable. In this case, the heat capacity curve C(β) was computed on a β-interval of ±0.1 with 50 equidistant points around the expected maximum from a finite-difference (second order forward) approach. $\beta_{\max}$ was then estimated from the smoothened profile via interpolation of the discretized function values using cubic splines.

For DOS calculations in case of periodic boundaries (PBC), we used the publicly available Mathematica-implementation [23], developed by Beale [12], based on Kaufman’s generalization [11] of Onsager’s solution [24]. For PBC, system sizes up to N=20 were considered. Note that all the derivations given in the following (such as the calculation of *C* and $\beta_{\max}$) are exact and general and hold for all types of boundary conditions in all dimensions; it is only the input in form of the DOS that differs for different systems.

Heat capacity profiles C(β) for the considered N×N-lattices were calculated based on the DOS from standard thermodynamic relations [25]:

(2) $$C=\partial_{T}\langle E\rangle =\beta^{2}\cdot \partial_{\beta \beta }\ln Z=\beta^{2}\underset{={\mathrm{Var}}_{E}}{\underset{︸}{\left(\langle E^{2}\rangle -{\langle E\rangle }^{2}\right)}}=\beta^{2}\;\frac{\sum_{n}{\left(E_{n}-\langle E\rangle \right)}^{2}\;\Omega_{n}\;e^{-\beta E_{n}}}{\sum_{n}\Omega_{n}\;e^{-\beta E_{n}}}$$

where 〈E〉 and $\langle E^{2}\rangle$ denote the first (i.e., the mean value) and second moment of the DOS, respectively. Though it is not explicitly represented for reasons of better readability, it should be noted that all moments $\langle E^{m}\rangle$ (see also Equation (5a)) and combinations thereof such as the energy variance ${\mathrm{Var}}_{E}$ are temperature-dependent (i.e., β-dependent). The dependency on system size *N* (either in the form of an explicit argument or as a subscript to emphasize its meaning as curve parameter) is only explicitly stated when it serves to improve comprehensibility. Throughout the paper, we use short-hand notations for (partial) derivative operators such as $\partial_{\beta }\equiv \left(\partial \ldots /\partial \beta \right)$ and $\partial_{\beta \beta }\equiv \left(\partial^{2}\ldots /\partial \beta^{2}\right)$ for the first and second partial derivatives, respectively (in this case with respect to β), while all other independent variables (such as $N_{\mathrm{spins}}$) are held constant.

Finding the (inverse) temperature ($\beta_{\max}$) at the peak maximum of the heat capacity profile follows from derivation of Equation (2):

(3) $$\partial_{\beta }C=2\beta \;{\mathrm{Var}}_{E}+\beta^{2}\;\partial_{\beta }{\mathrm{Var}}_{E}=C\cdot \left(\frac{2}{\beta }+\frac{\langle E\rangle \langle E^{2}\rangle -\langle E^{3}\rangle }{\langle E^{2}\rangle -{\langle E\rangle }^{2}}+2\;\langle E\rangle \right)$$

By setting the right-hand side to zero ($\partial_{\beta }C=0$), the problem of calculating $\beta_{\max}$ is reduced to finding the zeroes of the following function:

(4) $$f\left(\beta_{\max}\right)\equiv 2+\beta_{\max}\cdot \langle E\rangle \left(\beta_{\max}\right)\cdot \left(\frac{D(\beta_{\max})}{C(\beta_{\max})}+2\right)=0$$

with the supplementary relations

(5a) $$\begin{array}{cc} \langle E^{m}\rangle & =\frac{1}{Z}\sum_{n}E_{n}^{m}\;\Omega_{n}\;e^{-\beta E_{n}}=\frac{\sum_{n}E_{n}^{m}\;\Omega_{n}\;e^{-\beta E_{n}}}{\sum_{n}\Omega_{n}\;e^{-\beta E_{n}}} \end{array}$$

(5b) $$\begin{array}{cc} \partial_{\beta }\langle E^{m}\rangle & =\langle E^{m}\rangle \langle E\rangle -\langle E^{m+1}\rangle \end{array}$$

(5c) $$\begin{array}{cc} D & =\beta^{2}\left(\langle E^{2}\rangle -\frac{\langle E^{3}\rangle }{\langle E\rangle }\right) \end{array}$$

(5d) $$\begin{array}{cc} \partial_{\beta }D & =\frac{2D}{\beta }+\beta^{2}\left(\partial_{\beta }\langle E^{2}\rangle -\frac{\langle E\rangle \;\partial_{\beta }\langle E^{3}\rangle -\langle E^{3}\rangle \;\partial_{\beta }\langle E\rangle }{{\langle E\rangle }^{2}}\right) \end{array}$$

(5e) $$\begin{array}{cc} f(\beta ) & =2+\underset{=g}{\underset{︸}{\beta \;\langle E\rangle }}\cdot \underset{=h}{\underset{︸}{\left(\frac{D}{C}+2\right)}} \end{array}$$

(5f) $$\begin{array}{cc} f^{\prime }\left(\beta \right) & \equiv \partial_{\beta }f=h\cdot \partial_{\beta }g+g\cdot \partial_{\beta }h\\ \; & =\left(\langle E\rangle +\beta \cdot \partial_{\beta }\langle E\rangle \right)\cdot \left(\frac{D}{C}+2\right)+\beta \;\langle E\rangle \cdot \left(\frac{C\cdot \partial_{\beta }D-D\cdot \partial_{\beta }C}{C^{2}}\right) \end{array}$$

Obviously, for the Ising model featuring single-peaked heat capacity profiles, finding the maximum of C(β) is equivalent to finding the maximum of C(T), i.e., $C\left(\beta_{\max}\right)=\max\;C\left(\beta \right)=C_{\max}=\max\;C\left(T\right)=C\left(T_{\max}\right)$ with $\beta_{\max}=1/T_{\max}$. Since Equation (4) cannot be solved analytically and therefore no closed expression can be derived for $\beta_{\max}$, it was solved numerically via Newton’s method according to $\beta_{\max}^{\left(k+1\right)}:=\beta_{\max}^{\left(k\right)}-f\left(\beta_{\max}^{\left(k\right)}\right)/f^{\prime }\left(\beta_{\max}^{\left(k\right)}\right)$, using Equation (5e) and Equation (5f). $\beta_{\max}^{\left(k\right)}$ denotes the numeric value for $\beta_{\max}$ at the *k*-th iteration. Iterations were stopped once consecutive updates for $\beta_{\max}$ differed by less than $10^{-15}$ on an absolute scale and written to file with a precision of 14 decimal places. Numerical results for heat capacity peak data for FBC and PBC systems calculated by the approach described above are listed in the Appendix A in Table A1 and Table A2, respectively. It should be stressed that the only approximation involved in the procedure arises from the numerical determination of $\beta_{\max}$ from Equation (4).

While the maximum temperature is obtained from differentiation of C(β), another possible temperature measure that can be used as a proxy for the true critical temperature $T_{c}$ can be constructed from integration of the heat capacity curve. Changes in the mean energy U≡〈E〉 (for the Ising model, the thermodynamic concepts of (total) mean energy, internal energy, and potential energy coincide) and the entropy *S* between the two limiting temperature values of T=0 and T→∞ are related to integrals of C(T) and can be calculated analytically due to their state function property [18]. For FBC one obtains

(6a) $$\begin{array}{cc} \Delta U_{\mathrm{cal}} & =\int_{0}^{\infty }C\left(T\right)\;dT=\int_{0}^{\infty }\frac{C(\beta )}{\beta^{2}}d\beta =U\left(T\to \infty \right)-U\left(T=0\right)=2N\;\left(N-1\right) \end{array}$$

(6b) $$\begin{array}{cc} \Delta S_{\mathrm{cal}} & =\int_{0}^{\infty }\frac{C(T)}{T}\;dT=\int_{0}^{\infty }\frac{C(\beta )}{\beta }d\beta =S\left(T\to \infty \right)-S\left(T=0\right)=\left(N^{2}-1\right)\ln2 \end{array}$$

where the Jacobian factor of $-1/\beta^{2}$ for the transformation from *T*- to β-space has to be considered in the second step. Note that the integral of C(β) without the Jacobian factor would lack a direct thermodynamic interpretation. The subscript “cal” recalls the analogy to calorimetric experiments. The ratio of the two integral quantities introduces the corresponding calorimetric temperature which, in case of FBC, shows a N/(N+1)-scaling law with system size

(7) $$T_{\mathrm{cal}}\left(N\right)\equiv \frac{\Delta U_{\mathrm{cal}}}{\Delta S_{\mathrm{cal}}}=\frac{N}{N+1}\cdot \underset{=T_{\mathrm{cal}}\left(\infty \right)}{\underset{︸}{\frac{2}{\ln2}}}=\frac{3}{2}\frac{N}{N+1}\cdot T_{\mathrm{cal}}\left(2\right)\mathrm{for}N\geq 2$$

where the Binomial formula was applied in the first step and $T_{\mathrm{cal}}\left(2\right)=2/3\cdot T_{\mathrm{cal}}\left(\infty \right)=4/\left(3\ln2\right)$. It is evident that the limiting value of this temperature measure $T_{\mathrm{cal}}\left(\infty \right)=2/\ln2\approx 2.885$ is not identical to the true 2D-critical temperature $T_{c}=2/\ln\left(1+\sqrt{2}\right)\approx 2.269$ [19,24]. However, as it turns out, $T_{\max}\left(N\right)$ seems to be almost perfectly correlated with $T_{\mathrm{cal}}\left(N\right)$ (for FBC and PBC), implying that it obeys the identical N/(N+1)-scaling law (compare with the Section 3). Different interpretation approaches for $T_{\mathrm{cal}}$ are summarized in Appendix B.

Applying the same procedure for PBC, it is $\Delta U_{\mathrm{cal}}=2N^{2}$ instead of Equation (6a) due to the different value for the ground-state energy, yielding $T_{\mathrm{cal}}\left(N\right)=N^{2}/\left(N^{2}-1\right)\cdot 2/\ln2$ instead of Equation (7). However, since it was found that this approach does not deliver a satisfactory description of the data, it was not explored further. For the sake of completeness, it should be noted that if C(T) would be formally treated as a (non-normalized) probability density function, $T_{\max}$ would correspond to the mode of this distribution, and other characteristic distribution parameters, such as the mean and median temperatures, could be derived. However, since these parameters neither exhibit the same scaling behavior as $T_{\max}\left(N\right)$ (in contrast to $T_{\mathrm{cal}}\left(N\right)$) nor tend towards the true $T_{c}$, they are not further considered as relevant temperature measures.

In order to assess the quality of the presented approaches, we compared them in the case of FBC systems with the established cumulant intersection method proposed by Binder [10]. Therein, the fourth-order (“Binder”) cumulant $U_{L}$ is calculated according to

(8) $$U_{L}=1-\frac{\langle M^{4}\rangle }{3\;{\langle M^{2}\rangle }^{2}}$$

with the moments of the (instantaneous) magnetization $M\left(\left\{s_{i}\right\}\right)=\sum_{i=1}^{N_{\mathrm{spins}}}s_{i}$ according to

(9) $$\langle M^{m}\rangle =\frac{1}{Z}\sum_{j}M_{j}^{m}\;\Omega \left\{E\left(M_{j}\right)\right\}\;e^{-\beta E(M_{j})}=\frac{\sum_{j}M_{j}^{m}\;\Omega \left\{E\left(M_{j}\right)\right\}\;e^{-\beta E(M_{j})}}{\sum_{j}\Omega \left\{E\left(M_{j}\right)\right\}\;e^{-\beta E(M_{j})}}$$

where $\left\{s_{i}\right\}$ denotes an instantaneous spin configuration, $E(M_{j})$ is the energy level associated with the magnetization value $M_{j}$ and $\Omega \left\{E\left(M_{j}\right)\right\}=\Omega \left(E,M\right)$ corresponds to the joint DOS, i.e., the multiplicity of microstates with energy level *E* and magnetization *M*. In contrast to the energy-DOS introduced beforehand, this represents a 2D or joint DOS since it involves both energy and magnetization levels. The joint DOS was obtained exactly by two complementary numerical methods implemented in Python. For small lattices, we performed a full enumeration of all $\Omega_{\mathrm{tot}}$ configurations and accumulated counts into an (*E*, *M*)-histogram. For larger lattices, we used a TM/dynamic-programming extension that carries the row pattern and the running magnetization as state variables: row energies and row–row interaction energies were precomputed, and counts were propagated row-by-row while tracking both energy and magnetization. Results were stored as integer counts and validated by checking the closure relation $\sum_{E,M}\Omega \left(E,M\right)=\Omega_{\mathrm{tot}}=2^{N_{\mathrm{spins}}}$.

All implementations were performed in Python 3.11 using standard libraries (numpy, itertools, collections).

## 3. Results

In the following, the two basic extrapolation approaches studied in this work are presented. Modeled (i.e., approximated) quantities are indicated with a hat (such as ${\hat{T}}_{\max}$) to distinguish them notationally from the “true” estimates obtained from the DOS.

### 3.1. Modeling Maximum Heat Capacity Temperatures as a Function of System Size $T_{\max}\left(N\right)$

As already mentioned in the Section 2, we have found that the two temperature measures $T_{\max}$ and $T_{\mathrm{cal}}$ (compare Equation (7)) are not identical, i.e., they do not converge towards the same limiting value for N→∞ but they are correlated almost perfectly, i.e., they appear to follow the same scaling law with a N/(N+1) dependence on system size. This motivates the following approach with two model parameters:

(10a) $$\begin{array}{cc} {\hat{T}}_{\max}\left(N\right) & =a\cdot \frac{N}{N+1}+b \end{array}$$

(10b) $$\begin{array}{cc} \; & ={\hat{T}}_{c}-\frac{a}{N+1}\mathrm{with}{\hat{T}}_{c}=a+b \end{array}$$

In the equivalent formulation of Equation (10b), the target quantity ${\hat{T}}_{c}={\hat{T}}_{\max}\left(N\to \infty \right)$ appears as a direct model parameter. It is evident that when data are plotted as a function of inverse system size in the form of $\left\{{\left(N_{i}+1\right)}^{-1},T_{\max,i}\right\}$, a straight line can be fitted to the transformed data for which the slope and offset parameter correspond to the model parameters *a* and ${\hat{T}}_{c}$, respectively.

**Figure 1:**
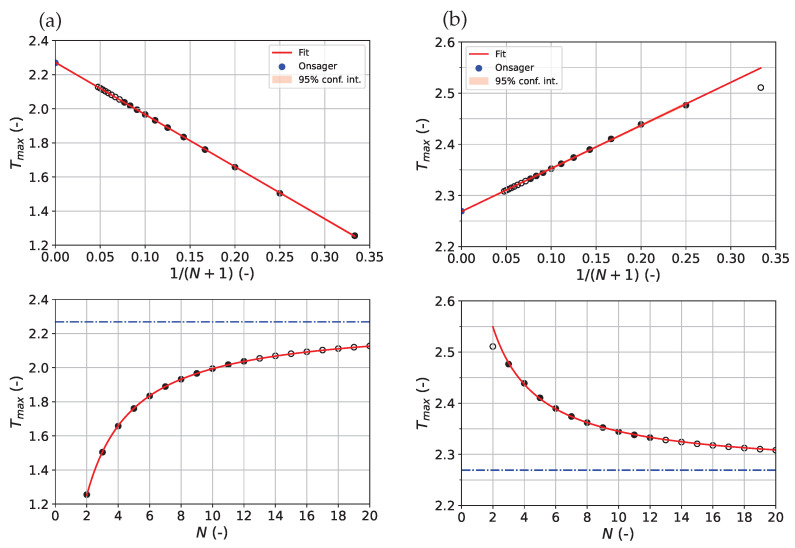
Dependence of the heat capacity peak temperature $T_{\max}$ as a function of system size up to N=20: FBC (**a**), PBC (**b**). Upper row: $T_{\max}$ over 1/(N+1) (linearized scale); lower row: $T_{\max}$ over *N* (native scale). Full circles correspond to calculated $T_{\max}$-values based on the DOS formalism that were included in the fitting procedure (2≤N≤12). Open circles represent calculations that were not included in the fitting procedure (13≤N≤20). For PBC, N=2 was also excluded from the fitting step (see main text). In the case of FBC, system sizes 13≤N≤20 (open circles) were not calculated directly from the DOS but from a finite differences approach (see Section 2). For both boundary types, fitting was performed using Equation (10b) on the linearized scale (red curve). The blue dashed–dotted horizontal line in the graphs of the lower row corresponds to the true critical temperature for N→∞ (“Onsager”). For the graphs of the upper row, the Onsager value is represented by the blue point on the *T*-axis. Note the different ranges for FBC and PBC on the *T*-axis. The 95% confidence interval of the modeled $T_{\max}$-values are represented but within a thickness of the fitting curve.

Figure 1 shows the excellent description of the heat capacity maximum temperature $T_{\max}$ as a function of system size *N* via Equation (10b) for both types of boundary conditions. System sizes 2≤N≤12 were used for the fitting step, following the described approach, while 13≤N≤20 was used as the test set for assessing model predictivity. In the case of PBC, it was found necessary to exclude the minimal 2×2-system from the fit (i.e., the smallest *N* included was N=3) to obtain a good description as shown in the plot. DOS-estimates for $T_{\max}$ were calculated iteratively using Equation (5e) and Equation (5f) as described in the Section 2. Key performance indicators to assess fitting quality and model predictivity are summarized in Table 1. As can be seen, Equation (10b) yields a relative deviation below ϵ<0.15% with respect to the true $T_{c}$. This is remarkable because only the information from the 11 smallest systems was taken into account for the adjustment. For PBC, the agreement is even better with ϵ<0.03%.

Using the high degree of correlation between the two temperature measures $T_{\max}$ and $T_{\mathrm{cal}}$, the presented approach can be simplified even further. Assuming direct proportionality in the form of $\Delta T_{\max}\left(N\right)=a\cdot \Delta T_{\mathrm{cal}}\left(N\right)$ with $\Delta T_{\max}\left(N\right)=T_{\max}\left(N\right)-T_{\max}\left(N_{\min}\right)$ and analogously $\Delta T_{\mathrm{cal}}\left(N\right)=T_{\mathrm{cal}}\left(N\right)-T_{\mathrm{cal}}\left(N_{\min}\right)$ with a freely selectable lower limit $N_{\min}$ leads to the following simplified relation:

(11) $${\hat{T}}_{\max}\left(N\right)={\hat{T}}_{c}\cdot \left(\frac{N-N_{\min}}{N+1}\right)+T_{\max}\left(N_{\min}\right)\cdot \left(\frac{N_{\min}+1}{N+1}\right)$$

where $a=\left(T_{\max}\left(\infty \right)-T_{\max}\left(N_{\min}\right)\right)/\left(T_{\mathrm{cal}}\left(\infty \right)-T_{\mathrm{cal}}\left(N_{\min}\right)\right)$. Equation (11) requires a single adjustable model parameter (${\hat{T}}_{c}$) and one additional $T_{\max}$ data point (from the DOS calculation) at some arbitrary size $N_{\min}$. Table 2 summarizes the model performance for the FBC systems using $T_{\max}$ for $N_{\min}=2$ as reference. We studied the impact of the number of considered systems that are included in the optimization process as denoted by the varying upper bound $N_{\max}$. Only systems $N_{\min}\leq N\leq N_{\max}$ were used for the fitting step, while systems $N_{\max}+1\leq N\leq 20$ were used for assessment of the model predictive power in terms of the mean absolute error (MAE). As can be seen, even with a single adjustable parameter, the predicted ${\hat{T}}_{c}$ is still very close to the the true $T_{c}$ as measured by the relative deviation ϵ in all cases. It is remarkable that in the extreme case where only the two smallest systems N={2,3} were used in the fitting step, ϵ is still below 1% and the predictive power for the other system sizes is still relatively good as measured by the small MAE value. For PBC, we obtain similar results.

### 3.2. Modeling Heat Capacity Maxima and Tangent Envelope

An alternative to modeling $T_{\max}$ as a function of system size *N* is to study the convergence of the sequence of heat capacity maxima $\left\{T_{\max,i},c_{\max,i}\right\}$ directly. For convenience, the maximum heat capacity is considered on a per-spin basis $c_{\max}=C_{\max}/N_{\mathrm{spins}}$. In Onsager’s classical paper [24], he already pointed out that the height of the specific heat peak grows with lnN, i.e., in the form of ${\hat{c}}_{\max}\left(N\right)=a\ln N+b$, which is in perfect agreement with our findings. Combining this with the known logarithmic singularity of the heat capacity in the vicinity of $T_{c}$ [5] motivates the following approach with three adjustable model parameters, including the estimate for the critical temperature ${\hat{T}}_{c}$:

(12a) $$\begin{array}{cc} {\hat{c}}_{\max}\left(T\right) & =a\ln\left(\frac{T}{{\hat{T}}_{c}-T}\right)+b\left(\mathrm{FBC}\right) \end{array}$$

(12b) $$\begin{array}{cc} {\hat{c}}_{\max}\left(T\right) & =a\ln\left(\frac{T}{T-{\hat{T}}_{c}}\right)+b\left(\mathrm{PBC}\right) \end{array}$$

The two slightly different expressions for the two boundary types (which differ in the argument of the logarithm) take into account that finite FBC-systems converge towards $T_{c}$ from $T_{\max}$-values, which are always lower than $T_{c}$, while finite PBC-systems approach it from higher temperatures (compare Figure 1, lower row).

**Figure 2:**
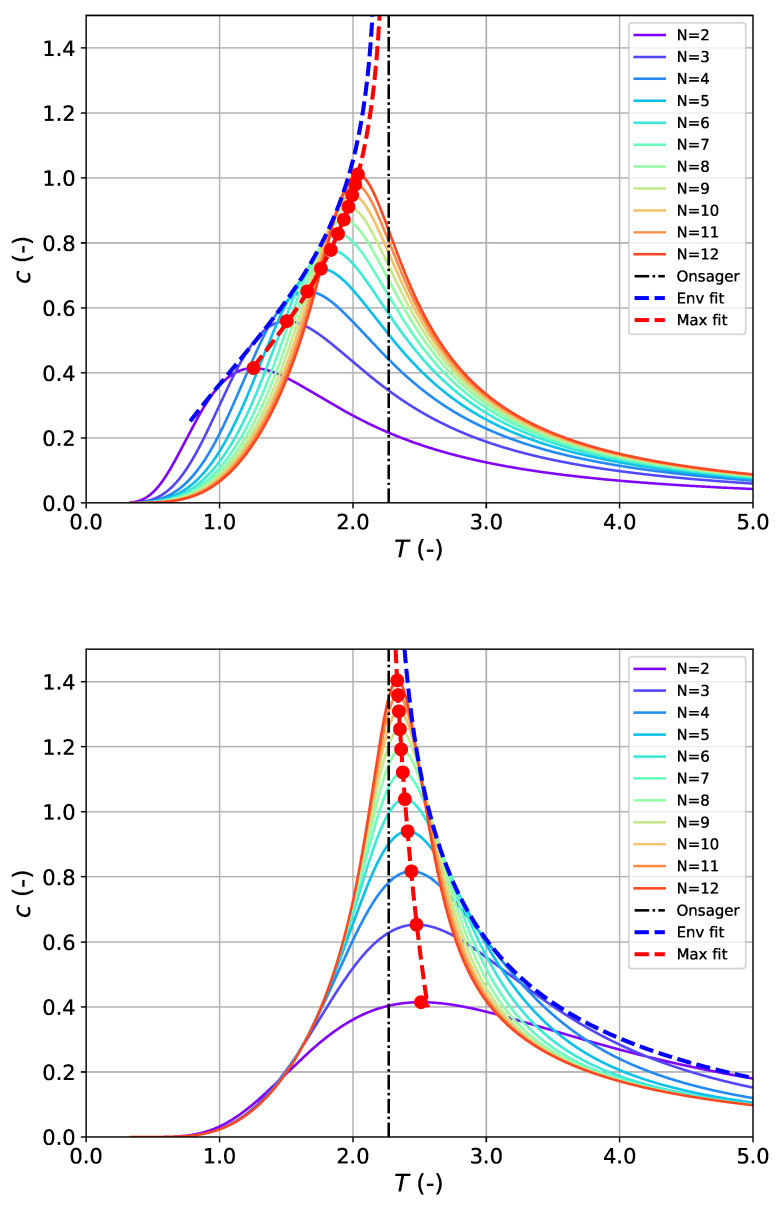
Heat capacity profiles (per spin) $c=C/N_{\mathrm{spins}}$ calculated via the DOS formalism as a function of temperature for system sizes up to N=12: FBC (**top**), PBC (**bottom**). Calculated heat capacity maxima are indicated by red circles. The dashed red and blue curves correspond to fits through the maxima and tangent points of the envelope, respectively. Fitting the maxima and the tangent envelope was performed using Equation (12a) in case of FBC (including system sizes 2≤N≤12) and Equation (12b) in case of PBC (including system sizes 3≤N≤12), respectively. The black dashed–dotted vertical line corresponds to the true critical temperature for N→∞ (“Onsager”).

In addition to the maxima, it seems plausible to examine the convergence of the envelope to the series of heat capacity profiles $c_{N}\left(T\right)$ as motivated from Figure 2, which shows $c_{N}\left(T\right)$ for 2≤N≤12. It can be assumed that in the thermodynamic limit, these envelopes should also converge towards the theoretical $T_{c}$-value. From the figure it becomes evident that for the set of FBC-curves, one tangent envelope should exist approaching $T_{c}$ from the left, i.e., low-temperature side, while for the set of PBC-curves, one tangent envelope should exist approaching $T_{c}$ from the right, i.e., high-temperature side. For consistency reasons, we applied the identical modeling approaches as for the maxima $c_{\max}\left(T\right)$ according to Equation (12) also for the construction of the tangent envelope $c_{\mathrm{env}}\left(T\right)$ (also separately for FBC and PBC). To construct the tangent envelope, standard tangency conditions were imposed: at each contact point $T_{i}$, the envelope $c_{\mathrm{env}}\left(T\right)$ must coincide with the corresponding curve, i.e., $c_{\mathrm{env}}\left(T_{i}\right)=c_{N}\left(T_{i}\right)$ as well as their corresponding derivatives $c_{\mathrm{env}}^{\prime }\left(T_{i}\right)=c_{N}^{\prime }\left(T_{i}\right)$ with $c^{\prime }\equiv \partial_{T}c$. The *T*-derivative $c_{N}^{\prime }\left(T\right)=\partial_{T}c_{N}\left(T\right)$ for the series of heat capacity curves calculated from the DOS is obtained from Equation (3) using the chain rule $\partial_{T}c_{N}\left(T\right)=\partial_{\beta }c_{N}\cdot d_{T}\beta =-1/T^{2}\cdot \partial_{\beta }c_{N}$. The derivate for the envelope $c_{\mathrm{env}}^{\prime }\left(T\right)$ is straightforward to obtain from Equation (12). This system of nonlinear equations was solved numerically using a least-squares minimization procedure, with bounds informed by the intersection points of adjacent curves.

Results of modeling quality are shown in Figure 2 and are summarized in Table 3. It can be seen that the overall deviation from the true $T_{c}$ based on ϵ is satisfactory but not as good as in the previous extrapolation strategy based on $T_{\max}\left(N\right)$, while the predictive power by the MAE is similar. A posteriori weighted averaging of the four individual estimates according to

(13a) $$\begin{array}{cc} \langle {\hat{T}}_{c}\rangle & =\frac{\sum_{n}w_{n}\;{\hat{T}}_{c,n}}{\sum_{n}w_{n}} \end{array}$$

(13b) $$\begin{array}{cc} \sigma \{\langle {\hat{T}}_{c}\rangle \} & =\frac{1}{\sqrt{\sum_{n}w_{n}}} \end{array}$$

with the weights $w_{n}=1/\sigma^{2}\left\{{\hat{T}}_{c,n}\right\}$ according to the individual inverse squared errors from Table 3 yields $\langle {\hat{T}}_{c}\rangle =2.2354\pm 0.0048$ and ϵ=1.4899%, which is not superior compared to the individual estimation results.

## 4. Discussion

In this work, we focused on extrapolation strategies based on the heat capacity profile of finite Ising lattices that are used as a proxy for the critical temperature of the infinite lattice $T_{c}$ and which we expect to converge towards $T_{c}$ for N→∞. While this is a common approach it is by no means unique. Another possible choice is to focus on the maximum of the zero-field susceptibility [26]. However, it appears that these different approaches do not necessarily lead to the same extrapolation result in practice due to the different underlying methods used for their determination or their limited accuracy [10]. For this reason, we have deliberately focused on the investigation of small systems for which the exact calculation of the partition function (or equivalently the DOS) is in principal possible, using suitable methods, even if no direct analytical expression can be given.

We have identified two approaches: “approach 1” is focused on modeling the maximum (or peak) temperature $T_{\max}\left(N\right)$ (c.f. Equation (10)); “approach 2” is focused on modeling the sequence of heat capacity maxima $\left\{T_{\max,i},c_{\max,i}\right\}$ together with (one-sided) tangent envelopes to the family of heat capacity profiles (c.f. Equation (12)). The appeal of approach 1 is that it involves a minimal number of adjustable parameters (in the simplified form of Equation (11), only 1), requires a minimum of exact data points from DOS calculations for adjustment, and can be converted into a linear representation, which allows straightforward and robust solution to the optimization problem. Further, it turns out that approach 1 can be applied to both types of boundary conditions and allows very accurate estimation of $T_{c}$ with a maximum relative deviation significantly below 1%. The results of approach 2 show a greater deviation from the true $T_{c}$ than approach 1, although the predictive power is similar. However, (slightly) different approaches must be chosen for FBC and PBC, each with three parameters, and a linear representation as for approach 1 is not possible, which makes optimization more challenging. An advantage is that, for a given boundary condition type, the same approach can be used to describe both the maxima and the common envelope. However, no method could be found to combine these partial results in a way that would yield a better estimate than the individual estimates. Overall, it can be said that approach 1 enables a more robust and accurate estimate for $T_{c}$.

In the case of PBC maxima, it was found that, for both tested extrapolation approaches to be necessary, the data from the smallest lattice with N=2 needed to be excluded from the modeling in order to obtain satisfactory results, which was not the case for FBC. This observation seems counterintuitive, as finite-size effects would be expected to be particularly pronounced in small FBC systems rather than PBC, and we are currently unable to provide an explanation for this.

**Figure 3:**
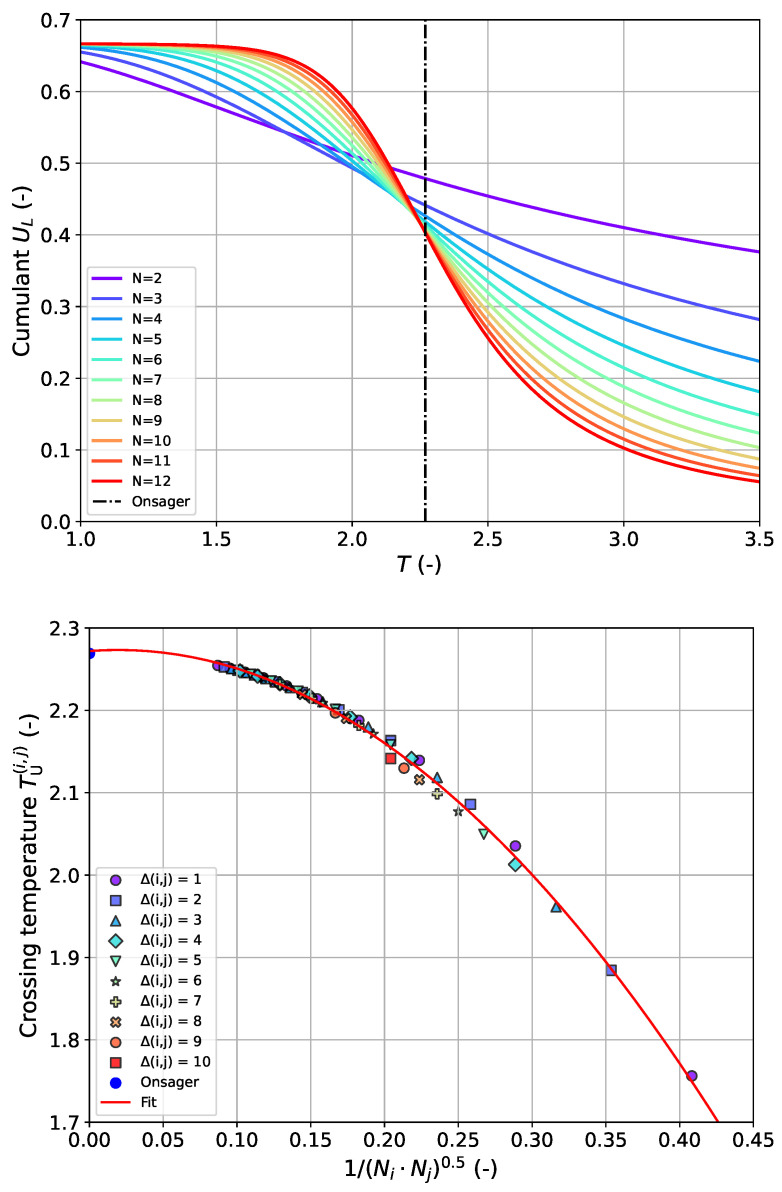
(**Top**) fourth-order (“Binder”) cumulant $U_{L}$ according to Equation (8) as a function of temperature for system sizes between $N_{\min}=2$ and $N_{\max}=12$ for FBC-lattices. The black dashed–dotted vertical line corresponds to the true critical temperature for N→∞ (“Onsager”). (**Bottom**) crossing temperatures $T_{U}^{\left(i,j\right)}$ determined from the intersection point between each pair ($N_{i}$, $N_{j}$) of $U_{L}$-curves for all $\left(N_{\max}-N_{\min}+1\right)\cdot \left(N_{\max}-N_{\min}\right)/2=55$ binary pairs as a function of inverse effective system size $1/N_{\mathrm{eff}}$ with $N_{\mathrm{eff}}={\left(N_{i}\;N_{j}\right)}^{0.5}$. The different symbols correspond to the difference in system size between *i* and *j* with $\Delta \left(i,j\right)=N_{j}-N_{i}$. The red line corresponds to the quadratic fit according to Equation (14). The Onsager value for the true critical temperature is represented by the blue point on the *T*-axis.

For further assessment of the accuracy of the presented approaches, we additionally estimated $T_{c}$ from the established cumulant intersection method [10] in case of FBC systems (calculation details can be found in the Section 2). The procedure is based on plotting the fourth-order (“Binder”) cumulant $U_{L}$ according to Equation (8) as a function of temperature for different system sizes and estimate $T_{c}$ from the common intersection point (denoted as crossing temperature $T_{U}^{\left(i,j\right)}$) between pairs of $U_{L}$-curves (*i*,*j*) [6]. In this way, a total of $\left(N_{\max}-N_{\min}+1\right)\cdot \left(N_{\max}-N_{\min}\right)/2=55$ values for $T_{U}^{\left(i,j\right)}$ is obtained with $N_{\min}=2$ and $N_{\max}=12$. It is known that the intersection points are scattered in the form of a pronounced size-dependence of the $T_{U}^{\left(i,j\right)}$-values especially in the case of very small systems, preventing an unambiguous determination of $T_{c}$. This effect can be seen in the top panel of Figure 3. In order to still be able to use the data from all pairs without excluding particular systems, we propose an evaluation by plotting over the inverse effective system size as given by the geometric mean of the sizes for each pair ($N_{\mathrm{eff}}={\left(N_{i}\;N_{j}\right)}^{0.5}$). We have found that all points fall onto a single curve to a very good approximation in that way (see lower panel of Figure 3). The simplest approach to modeling this relationship is a quadratic approach involving three parameters in the following form:

(14) $${\hat{T}}_{U}\left(x\right)=ax^{2}+bx+{\hat{T}}_{c}\mathrm{with}x=N_{\mathrm{eff}}^{-1}={\left(N_{i}\;N_{j}\right)}^{-0.5}$$

The estimate for the critical temperature ${\hat{T}}_{c}$ for the infinite lattice is obtained by extrapolation $N_{\mathrm{eff}}\to \infty$ which corresponds to the offset on the vertical axis in the lower graph of Figure 3. The success of this approach is shown in Figure 3 with the metrics listed in Table 4. As can be seen, the “Binder” cumulant method yields an ${\hat{T}}_{c}$ with similar accuracy as the corresponding estimate from approach 1; however, more adjustable model parameters are required.

It is important to clarify that the two presented approaches are not completely new (cf., e.g., Equation (4.1) in [27]), but only certain aspects. A novel aspect of our analysis that has not yet been discussed to the best of our knowledge is the observed strong correlation of $T_{\max}$ with the auxiliary quantity of the calorimetric temperature $T_{\mathrm{cal}}$. The temperature measure $T_{\mathrm{cal}}$ can be computed exactly from the thermodynamic properties of the Ising model in terms of energetic and entropic changes and, importantly, exhibits the correct scaling behavior with system size *N* according to N/(N+1). What both quantities have in common is that they can be calculated from the shape of the heat capacity profile C(T), while $T_{\max}$ is obtained from differentiation, i.e., the slope of C(T), $T_{\mathrm{cal}}$ is obtained from an integration procedure, i.e., the area under C(T) (although it can be calculated without the need to actually perform the numerical integration). In the broader thermodynamic context of heat transfer within thermodynamic cycles, $T_{\mathrm{cal}}$ is also known as the thermodynamic mean temperature $T_{m}$ [28] and can be interpreted as a characteristic average temperature of the system. Different interpretation approaches for $T_{\mathrm{cal}}$ (or $T_{m}$) are presented in Appendix B. Although the stringent physical/mathematical origin of the observed strong correlation between $T_{\mathrm{cal}}$ and the peak temperature $T_{\max}$ currently remains unclear, some analogy can be found in the field of protein thermodynamics. Here, one is interested in the energetic and entropic changes that occur during heat-induced (i.e., calorimetric) protein unfolding. The derivation is based on the Gibbs equation (which corresponds to the second approach presented in Appendix B) and considers protein unfolding as a kind of pseudo-phase transition from an ordered (folded) to a disordered (unfolded) state of the protein. Ref. [29] uses the example of lysozyme as the model protein to demonstrate that the midpoint temperature of the experimentally recorded heat capacity profile (i.e., $T_{\max}$) agrees very well with the enthalpy/entropy ratio (i.e., $T_{\mathrm{cal}}$). Since the experiments are conducted under constant-pressure rather than constant-volume conditions, calculation of $T_{\mathrm{cal}}$ involves the calorimetric enthalpy change rather than $\Delta U_{\mathrm{cal}}$, but otherwise, the definition is identical to Equation (7). As in the case of the finite Ising lattices, the transition between the two states (from “folded” to “unfolded”) takes place over a wide temperature range and C(T) (including $C_{\max}$) remains finite. The main reason behind the applicability of the analogy could be that in both cases, i.e., protein unfolding and finite Ising lattices, the cooperative unit is not made out of bulk material but is of finite size; in case of protein unfolding, the cooperative unit is determined by the size of the macromolecule [30] and in vitro protein experiments are typically conducted in highly diluted aqueous solutions, which is why the model of a single protein molecule dissolved in a background of low-molecular solvent often represents a relatively good approximation. This state of infinite dilution further resembles the situation of a single Ising lattice of finite size. Another similarity between the two problems is that the thermodynamic behavior of “typical” water-soluble proteins (such as lysozyme) can often be described to good approximation by rather simple statistical–mechanical models such as lattice models [31]. Within the assumptions of these models, each amino acid residue can, like a spin, only assume two discrete states (hydrophobic or polar).

It is also remarkable but not obvious why the identical approach works not only for FBC but also for PBC, while in the latter case, the corresponding scaling behavior that would follow in analogy according to $T_{\mathrm{cal}}\left(N\right)=N^{2}/\left(N^{2}-1\right)\cdot 2/\ln2$ (see Section 2) does not appear to be adequate. Nonetheless, as our results demonstrate, the scaling with system size as obtained from $T_{\mathrm{cal}}$ in the presented way can be employed as a robust estimator of the critical temperature. In the next step, the transferability to 3D lattices will be studied. Since practical calculation of the exact DOS is limited to N={2,3} in 3D, a sophisticated DOS estimator such as the Wang–Landau method [32,33] has to be applied. Another new aspect of the present work is the further simplified variant given by Equation (11) with only one model parameter. In approach 2, the description of the envelope and the fact that it can be described to a good approximation using the same function as for the maxima curve are, to the best of our knowledge, also new.

In our previous work [18], we posed the central question whether it is possible to approximate a finite-sized 2D-Ising lattice via 1D Ising chains on the basis of free energy expressions. The approach involved a combination of an exact analytical intra-chain and an approximative inter-chain part through which interactions among adjacent chains are established. While the free energy contribution of these inter-chain bonds could be well approximated as a function of temperature and system size, unfortunately, it has been found that the extrapolation behavior N→∞ is not possible without imposing the target solution for $T_{c}$ in the form of an additional constraint into the model. With the relatively simple scaling law for $T_{\max}\left(N\right)$ presented in this work, we hope to be able to improve the approach of our previous work in future studies by incorporating it into the free energy construction scheme.

## Figures and Tables

**Table 1:** Analysis of modeling performance for $T_{\max}\left(N\right)$ according to Equation (10b). Model parameters were optimized from fitting to DOS-estimates of $T_{\max}$ for system sizes 2≤N≤12 in case of FBC and 3≤N≤12 for PBC. Fitting was performed using the linear representation of the data according to the upper row of Figure 1. Different metrics for assessing the quality of the fit and the model prediction capability are given: (i) predicted ${\hat{T}}_{c}$ together with the second model parameter *a*; (ii) relative percentage error between predicted and true $T_{c}$ according to $\epsilon \left(\%\right)=|{\hat{T}}_{c}-T_{c}|/T_{c}\cdot 100\%$ with $T_{c}=2/\ln\left(1+\sqrt{2}\right)$; (iii) coefficient of determination ($R^{2}$) and root mean square error (RMSE) refer to the quality of the fitting step; (iv) mean absolute error (MAE) towards the model prediction was evaluated for the test set of system sizes 13≤N≤20, which were not included for parameter optimization to assess the model predictive power.

Type	${\hat{T}}_{c}$	*a*	ϵ (%)	$R^{2}$	RMSE	MAE
FBC	2.2719±0.0012	3.0597±0.0071	0.1216	1.0000	0.0016	$1.318\;\times \;10^{-3}$
PBC	2.2686±0.0012	−0.8418±0.0082	0.0267	0.9992	0.0012	$5.692\;\times \;10^{-4}$

**Table 2:** Analysis of modeling performance for $T_{\max}\left(N\right)$ according to Equation (11) applied for FBC with $N_{\min}=2$ and $T_{\max}\left(N_{\min}=2\right)\approx 1.25546$ from DOS calculation (see Table A1). The single model parameter was optimized from fitting to DOS-estimates of $T_{\max}$ for system sizes $N_{\min}\leq N\leq N_{\max}$ with varying upper bound $N_{\max}$ as given in the first column. Different metrics for assessing the quality of the fit and the model prediction capability are given: (i) predicted ${\hat{T}}_{c}$; (ii) relative percentage error between predicted and true $T_{c}$ according to $\epsilon \left(\%\right)=|{\hat{T}}_{c}-T_{c}|/T_{c}\cdot 100\%$ with $T_{c}=2/\ln\left(1+\sqrt{2}\right)$; (iii) coefficient of determination ($R^{2}$) and root mean square error (RMSE) refer to the quality of the fitting step; (iv) mean absolute error (MAE) towards the model prediction was evaluated for the test set of system sizes $N_{\max}+1\leq N\leq 20$, which were not included for parameter optimization to assess the model predictive power.

$N_{\max}$	${\hat{T}}_{c}$	ϵ (%)	$R^{2}$	RMSE	MAE
12	2.2700±0.0013	0.0368	0.9999	0.0021	$2.313\;\times \;10^{-3}$
10	2.2691±0.0014	0.0045	0.9999	0.0021	$2.939\;\times \;10^{-3}$
8	2.2675±0.0018	0.0724	0.9999	0.0021	$3.967\;\times \;10^{-3}$
5	2.2623±0.0028	0.3038	0.9999	0.0017	$7.428\;\times \;10^{-3}$
3	2.2509±0.0000	0.8057	1.0000	0.0000	$1.488\;\times \;10^{-2}$

**Table 3:** Analysis of modeling performance for c(T) in the form of maxima (Max) and tangent envelope (Env) according to Equation (12). Model parameters were optimized from fitting to system sizes 2≤N≤12. In case of PBC maxima, the smallest size N=2 was excluded from optimization. Fitting the maxima as well as the tangent envelope was performed using Equation (12a) in case of FBC and Equation (12b) in case of PBC, respectively (see also Figure 2). Different metrics for assessing the quality of the fit and the model prediction capability are given as follows: (i) predicted ${\hat{T}}_{c}$ together with the model parameters *a* and *b*; (ii) relative percentage error between predicted and true $T_{c}$ according to $\epsilon \left(\%\right)=|{\hat{T}}_{c}-T_{c}|/T_{c}\cdot 100\%$ with $T_{c}=2/\ln\left(1+\sqrt{2}\right)$; (iii) coefficient of determination ($R^{2}$) and root mean square error (RMSE) refer to the quality of the fitting step; (iv) mean absolute error (MAE) towards the model prediction was evaluated for the test set of system sizes 13≤N≤20 which were not included for parameter optimization to assess the model predictive power.

Type	${\hat{T}}_{c}$	*a*	*b*	ϵ (%)	$R^{2}$	RMSE	MAE
FBC-Max	2.2383±0.0095	0.2844±0.0054	0.3495±0.0025	1.3606	0.9998	0.0027	$6.730\;\times \;10^{-3}$
FBC-Env	2.1874±0.0083	0.2700±0.0031	0.4099±0.0019	3.7219	0.9999	0.0016	n.a. ^1^
PBC-Max	2.1867±0.0190	1.1768±0.1230	−1.8632±0.1912	3.6372	0.9994	0.0056	$3.761\;\times \;10^{-2}$
PBC-Env	2.2835±0.0076	0.5205±0.0099	−0.1377±0.0085	0.6328	0.9998	0.0045	$1.858\;\times \;10^{-3}$

^1^ Envelope construction for N>12 in case of FBC does not follow from the DOS formalism, since here the external code of [21] has been used (see Section 2).

**Table 4:** Analysis of modeling performance according to Equation (14) for the data of crossing temperatures $T_{U}^{\left(i,j\right)}$ obtained from the cumulant intersection method for FBC systems with 2≤N≤12 (see also Figure 3). Different metrics for assessing the quality of the fit and the model prediction capability are as follows: (i) predicted ${\hat{T}}_{c}$ together with the model parameters *a* and *b*; (ii) relative percentage error between predicted and true $T_{c}$ according to $\epsilon \left(\%\right)=|{\hat{T}}_{c}-T_{c}|/T_{c}\cdot 100\%$ with $T_{c}=2/\ln\left(1+\sqrt{2}\right)$; (iii) coefficient of determination ($R^{2}$) and root mean square error (RMSE) refer to the quality of the fitting step. For comparison: the crossing temperature obtained from direct intersection between the two largest systems N={11,12} is 2.2545.

${\hat{T}}_{c}$	*a*	*b*	ϵ (%)	$R^{2}$	RMSE
2.2721±0.0055	−3.4641±0.1255	0.1341±0.0554	0.1272	0.9962	0.0059

## Data Availability

The simulation and analysis scripts presented in this study are available on request from the main author.

## Abbreviations

The following abbreviations are used in this manuscript:

DOS	Density of states
FBC	Free boundary conditions
MAE	Mean absolute error
MC	Monte Carlo
PBC	Periodic boundary conditions
RMSE	Root mean square error
TM	Transfer-matrix

## Appendix A

**Table A1:** Calculated heat capacity peak data ($\beta_{\max}$, $T_{\max}$, $c_{\max}$, all dimensionless) via the DOS formalism as described in the Section 2 in case of FBC systems for system sizes 2≤N≤12.

N	$\beta_{\max}$	$T_{\max}$	$c_{\max}$
2	0.79652072691636	1.25546011071348	0.41477832427870
3	0.66475179681815	1.50432086800897	0.55941445226867
4	0.60321817707788	1.65777497761128	0.65089277358858
5	0.56797741359190	1.76063339152165	0.72069049552003
6	0.54519688257892	1.83419977617948	0.77831036675863
7	0.52927443338945	1.88937900059907	0.82792070481326
8	0.51752465400304	1.93227509504142	0.87176111804541
9	0.50850125620929	1.96656348000922	0.91119481873195
10	0.50135661110095	1.99458823890655	0.94712420447240
11	0.49556112888914	2.01791452497821	0.98018354839189
12	0.49076693049847	2.03762710536404	1.01083907424538

**Table A2:** Calculated heat capacity peak data ($\beta_{\max}$, $T_{\max}$, $c_{\max}$, all dimensionless) via the DOS formalism as described in the Section 2 in case of PBC systems for system sizes 2≤N≤20.

N	$\beta_{\max}$	$T_{\max}$	$c_{\max}$
2	0.39826036345818	2.51092022142696	0.41477832427870
3	0.40379904431248	2.47647936290349	0.65331944163238
4	0.41001245261703	2.43895031386775	0.81646116140828
5	0.41484129044634	2.41056043125330	0.93962357730314
6	0.41846082094576	2.38970997987317	1.03846616661979
7	0.42121828956349	2.37406595292028	1.12100058870542
8	0.42337367711912	2.36197962708634	1.19183881979597
9	0.42510039070010	2.35238551146260	1.25387739667353
10	0.42651344202428	2.34459199047487	1.30905540966374
11	0.42769079897607	2.33813774435664	1.35873502267216
12	0.42868680203230	2.33270535798921	1.40390977092745
13	0.42954036009796	2.32806993915997	1.44532654684125
14	0.43028002213607	2.32406792914907	1.48356096157353
15	0.43092719410525	2.32057761422166	1.51906596488409
16	0.43149823532850	2.31750658085238	1.55220436002469
17	0.43200586061338	2.31478341191981	1.58327121815459
18	0.43246010078624	2.31235204862123	1.61250973691218
19	0.43286897518268	2.31016787372665	1.64012271652440
20	0.43323897181035	2.30819493412921	1.66628103025601

## Appendix B

In the following, different approaches for the interpretation of the temperature scale $T_{\mathrm{cal}}$ as introduced via Equation (7) are presented and the equivalence to the “thermodynamic mean temperature” $T_{m}$ as known from technical thermodynamics is demonstrated.

One approach originates from phenomenological technical thermodynamics, whereby two different processes are considered for a system in thermal exchange with its environment: (i) The real process I, in which a certain amount of heat *Q* is transferred into the system at variable temperature between initial temperature $T_{1}$ and final temperature $T_{2}$. This corresponds to the situation described by Equation (6a) and Equation (6b) where the lattice is heated up from $T_{1}=0$ to $T_{2}\to \infty$ while monitoring the change in heat capacity C(T). (ii) An isothermal reference process II, in which the same amount of heat is transferred at unique, i.e., constant, temperature $T_{m}$. $T_{m}$ lies between $T_{1}$ and $T_{2}$, and corresponds to that particular temperature at which the same entropy change ΔS is achieved as in the real process I, i.e., in terms of the second law:

(A1) $$\Delta S=\underset{I}{\underset{︸}{\int_{1}^{2}\frac{dQ}{T}}}=\underset{II}{\underset{︸}{\frac{Q}{T_{m}}}}$$

where for both processes, *Q* is assumed to be transferred reversibly and is given according to the first law by Q=ΔU since no work is performed. As can be seen, $T_{m}$ corresponds to the temperature at which Equation (A1) is satisfied (i.e., identical ΔU and ΔS for both processes), i.e., *Q* is transferred at uniform temperature but with the same effect as in the real process I. The definition above is suitable, for example, in thermodynamic cyclic processes where heat flows are transferred at non-constant temperatures to still make it possible to calculate the Carnot efficiency [28].

A second more molecular-based approach starts from the Gibbs equation for the Helmholtz free energy change according to ΔA=ΔU−TΔS. For a phase equilibrium between two phases or the transition from one phase to the other, respectively, taking place at constant temperature $T_{m}$, ΔU and ΔS measure the corresponding changes in internal energy and entropy, respectively, associated with the phase transition and ΔA=0, i.e., $T_{m}{|}_{\Delta A=0}=\Delta U/\Delta S$ (where the notation | means “under the condition”). This corresponds to the situation for a conventional phase transition such as the solid-to-liquid transition in case of melting of ice where the solid phase melts at constant melting temperature $T_{m}$. The finite systems studied in this work however do not feature a phase transition in the classic sense: the cooperative unit is not bulk material and the heat capacity peak is lacking a singularity but stays finite over the whole temperature range. The mean (or calorimetric) temperature defined through Equation (7) can be viewed as an equivalent temperature (in the sense of equivalent to the melting point for bulk systems) which mimics the situation of a classic phase transition occurring at uniform temperature where ΔU and ΔS are calculated over the whole (fixed) temperature interval from $T_{1}=0$ to $T_{2}\to \infty$ where the “transition” of the finite system (as judged by the heat capacity profile C(T)) takes place, according to Equation (6a) and Equation (6b). Note that since the Gibbs equation above was derived from the fundamental equation in the form A(T,V) (for a one-component system with constant number of particles and system volume *V*), it combines the first and second law for a reversible process, i.e., the two presented approaches are, of course, equivalent.

For the sake of completeness, a more formal interpretation for $T_{\mathrm{cal}}$ can be derived by rewriting Equation (7) in the following form:

(A2) $$T_{\mathrm{cal}}=\frac{\int_{U(0)}^{U(\infty )}dU}{\int_{S(0)}^{S(\infty )}dS}=\frac{\int_{S(0)}^{S(\infty )}T\;dS}{\int_{S(0)}^{S(\infty )}dS}=\frac{\int_{0}^{\infty }T\;\frac{C(T)}{T}\;dT}{\int_{0}^{\infty }\frac{C(T)}{T}\;dT}=\frac{\int_{0}^{\infty }T\;p\left(T\right)\;dT}{\int_{0}^{\infty }p\left(T\right)\;dT}={\langle T\rangle }_{p}$$

In the second step, the fundamental equation in the form of U(S,V) for a one-component system with constant number of particles, dU=TdS−PdV, was applied, assuming constant system volume *V* (i.e., an isochoric change) as appropriate for the processes and systems of interest, since no pressure (P)-volume (V) work is performed. The second equality typically represents the definition of the thermodynamic mean temperature $T_{m}$ in textbooks of technical thermodynamics. From this definition, it becomes immediately clear that although $T_{m}$ lies between the two temperatures of the integration limits, it is typically not a simple arithmetic mean of these values. In the third equality, the relationship dS=C(T)/TdT has been applied. Equation (A2) further illustrates that if we interpret p(T)=C(T)/T as a non-normalized probability density function as a function of temperature, $T_{\mathrm{cal}}$ formally corresponds to the first moment (i.e., mean) with respect to p(T).
